# A Data Envelopment Analysis Evaluation Study of Urban Crowd Sourcing Competitiveness Based on Evidence From 21 Chinese Cities

**DOI:** 10.3389/fpsyg.2022.861841

**Published:** 2022-03-25

**Authors:** Xiangdong Shen, Yixian Gu, Xinyou Zhao, Jingwen Xu

**Affiliations:** ^1^Business School, Changshu Institute of Technology, Suzhou, China; ^2^Suzhou Agricultural Modernization Research Center, Suzhou, China

**Keywords:** service outsourcing, crowd sourcing, DEA, competitiveness, evaluation study

## Abstract

In the era of the global village, crowd sourcing as a new model of service outsourcing is increasingly being valued by all walks of life. This study uses the data envelopment analysis (DEA) method to explain the crowd sourcing competitiveness of service outsourcing base cities by using input-output efficiency. The crowd sourcing competitiveness among crowd sourcing base cities is organized and analyzed by collating and analyzing the data of 21 service outsourcing base cities in China from 2016 to 2019. The results show that there is no significant difference in the competitiveness of 21 service outsourcing, the overall trend is that the competitiveness of crowd sourcing is not strong, the match between input and output is not high. Comparatively speaking, Beijing, Shanghai, Nanjing, Hangzhou, Suzhou and Wuxi are more competitive in crowd sourcing. Combining with the reality of China's economic development, taking the road of crowd sourcing with Chinese characteristics is a good choice for the development of China's crowd sourcing industry.

## Introduction

The individuation of customer demand is destined to gradually increase the gap between the enterprise's traditional innovation mode focusing on internal operation and the market (Modaresnezhad et al., 2020; Wu and Gong, 2021). Eric pointed out that users are innovators, and with the popularity of the Internet, this trend will become more and more obvious (Eric, 1988). In the masterpiece “The world is flat,” Friedman pointed out that enterprises complete non-core businesses with the help of external forces through outsourcing (Friedman, 2006). Wired magazine published an article by Jeff Howe, which first mentioned the concept of crowd sourcing, which is a new model for the development of service outsourcing. Howe pointed out that enterprises, institutions and even individuals outsourced the work tasks performed by employees to non-specific social mass groups in a free and voluntary manner (Howe, 2006). Crowd sourcing changes the focus of enterprise innovation model from internal operation to external acquisition. More and more enterprises realize the important value of crowd sourcing. IBM, PROCTOR, GEMBLE, BOEING and other Fortune 500 enterprises have improved their competitiveness through crowd sourcing (Shergadwala et al., 2020; Ziegler et al., 2021). The advent of the era of Globalization 3.0 promotes the rapid development of crowd sourcing mode. P & G has more than 9000 R & D personnel, but there are as many as 1.5 million external R & D personnel with the help of crowd sourcing. The main body of crowd sourcing mode includes the employer, the contractor and the intermediary. The process of crowd sourcing is generally divided into four main stages: task contracting, crowd sourcing task application, crowd sourcing task execution and crowd sourcing task completion and submission (Hou et al., 2020; Gordon, 2021). The causes of crowd sourcing are many and complex. Through literature review, it is found that scholars generally believe that the rise of the Internet, reducing costs, enhancing information symmetry, encouraging customer participation and enterprise crisis awareness are the main factors.

In recent years, crowd sourcing has been increasingly valued by the corporate world, but its research in academia is still in its infancy. Research scholars include Mandera et al. (2020), Shergadwala et al. (2020) and Deichmann et al. (2021). Through analysis, it is found that the existing research has two shortcomings: first, the research methods are mostly qualitative analysis and case description, lacking the support of empirical data; second, the research perspective directly applies the foreign crowd sourcing model to Chinese enterprises, lacking the links to the practical background of China's service outsourcing industry. Based on this, this study uses the DEA method to conduct quantitative research on the competitiveness evaluation of crowd sourcing model in 21 service outsourcing base cities, aiming to find a suitable way for the development of China's crowd sourcing industry.

The first purpose of this study is to conduct a quantitative study on the city data of 21 service outsourcing bases in China through the DEA method. The second purpose is to find the problems of crowd sourcing industry in China's main service outsourcing cities through DEA analysis. The third purpose is to provide countermeasures and suggestions for the development of crowd sourcing industry in China.

## Research Methods and Variable Selection

### Research Methods

Data envelopment analysis (DEA) is an efficiency evaluation method proposed by the famous American operations researcher Charnes et al. (1978). The DEA method is an effective method to evaluate the efficiency of a class of decision making units (DMU) with multiple inputs and multiple outputs. With the help of mathematical programming, the DMU is projected onto the front surface of DEA, and the degree of deviation of the DMU from the front surface of DEA is compared to evaluate its relative effectiveness (Dyckhoff and Allen, 2001).

There are “n” service outsourcing base cities, namely decision-making units (DMU), each DMU has “m” input variables (Xj) and “s” output variables (Yj), V and U are weights, Xj = (X1j, X2j, …, Xmj)T, Yj = (Y1j, Y2j, …, YSj)T, V = (V1, V2,…, VM)T, U = (U1, U2,…, US)T, Xij >0, Yri > 0, Vi > 0, Ur > 0, j = 1, 2, …, n; i = 1, 2, …, s. Xij is the input quantity of the ith input variable of the jth DMU, and Yrj is the output quantity of the rth output variable of the jth DMU. Then the competitiveness evaluation index of the jth DMU is:

Ej = $\frac{{\text{U}}^{\text{T}}{\text{Y}}_{\text{j}}}{{\text{V}}^{\text{T}}{\text{X}}_{\text{j}}}$, j = 1,2,…,n. For the jth DMU, according to the DEA basic model C2R, there are:


$$\begin{array}{c} |\begin{array}{c} \text{min }\theta =\theta^{*}\\ s.t.\sum \lambda_{j}X_{j}\leq \theta X_{0}\\ \sum_{j=1}^{n}\lambda_{j}Y_{j}\geq {\text{Y}}_{0}\\ \lambda j\geq 0\\ j=1,2,\ldots ,n \end{array};|\begin{array}{c} \max\;\delta =\delta^{*}\\ s.t.\sum \lambda_{j}X_{j}\leq X_{0}\\ \sum_{j=1}^{n}\lambda_{j}Y_{j}\geq \;\delta {\text{Y}}_{0}\\ \lambda_{j}\geq 0\\ j=1,2,\ldots ,n \end{array} \end{array}$$

Using Charnes-Cooper fractional transformation and the dual theory of linear programming, and introducing slack variables S+ and S-, which represent the amount of output that can be increased and the amount of input that can be decreased, the dual programming model is:


$$\begin{array}{c} |\begin{array}{c} \text{min }\theta =\theta^{*}\\ s.t.\sum_{i=1}^{n}\lambda_{i}X_{i}+s^{-}=\theta X_{0}\\ \sum_{i=1}^{n}\lambda_{i}Y_{i}-s^{+}={\text{Y}}_{0}\\ \lambda_{i}\geq 0,j=1,2,\ldots ,n\\ s^{-}\geq 0,s^{+}\geq 0 \end{array};|\begin{array}{c} \max\;\delta =\delta^{*}\\ s.t.\sum_{j=1}^{n}\lambda_{j}X_{j}+s^{-}\\ \sum_{j=1}^{n}\lambda_{j}Y_{j}-s^{+}\geq \;\delta {\text{Y}}_{0}\\ \lambda_{j}\geq 0,j=1,2,\ldots ,n.\\ s^{-}\geq 0,s^{+}\geq 0 \end{array} \end{array}$$

According to DEA theory, if the optimal solution of the j_0_th DMU is θ^*^, λ^*^, S^*+^, and S^*−^, there are: (1) θ^*^ = 1 and S^*−^ = 0, S^*+^ = 0, then the decision-making unit j_0_ is DEA effective, and the DMU economic activity is both technology and scale effective; (2) θ^*^ = 1 and S^*−^ > 0 or S^*+^ > 0, then the decision-making unit j_0_ is weakly DEA effective, and the DMU economic activities are non-simultaneously technical and scale effective; (3) θ^*^ < 1, the decision-making unit j0 is not valid for DEA; (4) If there is $\lambda_{\text{j}}^{*}$ (j = 1, 2, …, n) such that $\sum \lambda_{\text{j}}^{*}$ = 1, then DMU has constant returns to scale; if $\sum \lambda_{\text{j}}^{*}$ < 1, then DMU has increasing returns to scale; if $\sum \lambda_{\text{j}}^{*}$ > 1, then DMU has diminishing returns to scale.

### Variable Selection

#### The Ratio of the Number of Internet Users X_1_

The crowd sourcing model is a new type of service outsourcing model based on the Internet. It faces many discrete resource groups around the world. Participants who work in their spare time around the world gather in the community to provide services through the Internet. The popularity of the Internet provides a material basis for the development of crowd sourcing, eliminating the gap between time and space (Nevo and Kotlarsky, 2020). Network technology provides technical conditions for crowd sourcing (Li et al., 2021). Crowd sourcing in the Internet age is the real crowd sourcing (Donlon et al., 2020). Based on these considerations, this study takes the ratio of the number of Internet users in base cities to the number of Internet users in the country as an input variable (X_1_) of the DEA model.

#### The Ratio of the Number of Colleges X_2_

Crowd sourcing means the transfer and release of innovation inside the enterprise to the outside. Public participation in innovation brings new vitality to the adoption of crowd sourcing by enterprises. Innovation requires certain knowledge and skills, and new knowledge and skills can be learned through crowd sourcing (Brabham, 2008). Crowd sourcing requires group wisdom and a certain level of knowledge, which can be solved by people with different knowledge and skills (Sawhney and Prandell, 2001). Generally speaking, the gathering place and birthplace of knowledge and skills are colleges and universities, and the resources of colleges and universities have a lot of time to apply for crowd sourcing tasks and execute them (Xu et al., 2022). Based on these considerations, this study takes the ratio of the number of universities in base cities to the number of universities in the country as an input variable (X_2_) of the DEA model.

#### The Ratio of the Number of Registered Enterprises X_3_

As the main contractor of the crowd sourcing model, the enterprise publishes the crowd sourcing tasks to the outside world, and completes the innovation activities of the enterprise with the help of the wisdom of the outside world through the Internet. The Chinese government strongly encourages enterprises to transform and upgrade, and strive to transform from “Made in China” to “Created in China.” More and more enterprises in China are completing innovation activities through crowd sourcing. For example, Geely Automobile collects logos from all over the world, Haier collects design ideas from users, Huawei mobile phones of some components come from many countries around the world. When many companies use the crowd sourcing model to complete innovation activities, the atmosphere of “Created in China” will be stronger (Shao et al., 2012; Jaribion et al., 2021). Based on these considerations, this study takes the ratio of the number of registered enterprises in base cities to the number of registered enterprises in the country as an input variable (X_3_) of the DEA model.

#### Enterprise Asset Size Ratio X_4_

Large enterprises are always at the forefront of innovation and reform, and enterprises with strong assets and large scale are always aware of crowd sourcing. For example, IBM has invested 1 billion US dollars to develop a crowd sourcing model suitable for its own enterprise; BMW has opened a customer innovation laboratory, using the crowd sourcing model to provide users with online tools to help them participate in the innovative design of BMW cars (Füller et al., 2021). These companies are committed to adopting advanced business models to occupy the commanding heights of the market, and continue to complete innovative activities through the crystallization of the wisdom of the crowdsourced Haina group (Erica et al., 2020). Based on these considerations, this study takes the ratio of the base city's enterprise asset scale to the national enterprise asset scale as an input variable (X_4_) of the DEA model.

#### The Ratio of the Number of Intermediaries X_5_

The intermediary plays the role of building a bridge of communication between the contracting party and the contracting party. For example, AMAZON launched Mechanical Turk (Beta version), a platform for providing crowd sourcing services (Shank, 2016), INNOCENTIVE uses a competition-based online crowd sourcing innovation model platform to help multinational companies solve scientific and engineering problems (Welinder and Perona, 2010). Crowd sourcing intermediaries can assist the government in researching and formulating crowd sourcing industry policies and regulations, and can also encourage users to maintain the quality of service delivery to meet the quality requirements of enterprises. In addition, crowd sourcing intermediaries are also committed to safeguarding the intellectual property rights and information security of enterprises, and cooperating with contracting parties to formulate crowd sourcing industry strategies (Donlon et al., 2020). Based on these considerations, this study takes the ratio of the number of intermediaries in base cities to the number of intermediaries in the country as an input variable (X_5_) of the DEA model.

#### The Ratio of the Number of Regulations Related to Service Outsourcing Issued X_6_

China has successively introduced a number of support policies for the service outsourcing industry, including finance and taxation, talent training, employment of college students, special working hours, customs supervision, telecommunications services, financial support, intellectual property protection, and investment promotion (Cuccolo et al., 2020; Ullah et al., 2021). After nearly 30 years of hard work, China's intellectual property legal system has gradually improved. There are more than 20 laws and regulations related to intellectual property issued in China, and many regulations on information security management have also been issued, all of which have promoted service outsourcing intellectual property rights. Good development of protection and information security management. In addition, local governments in China have also issued a number of laws and regulations related to service outsourcing, aiming to promote the transformation and upgrading of the service outsourcing industry and achieve leapfrog development (Ozcan et al., 2020). Based on these considerations, this study takes the ratio of the number of regulations related to service outsourcing issued in base cities to the number of regulations related to outsourcing issued in China as an input variable (X_6_) of the DEA model.

#### Corporate Revenue Ratio Y_1_

To a certain extent, corporate income reflects the business performance of the company. Theoretically, under the same conditions, the income obtained by the enterprise through the crowd sourcing model, with the help of the wisdom of the outside group, should be greater than the income from innovation behind closed doors (Dissanayake et al., 2021; Lim et al., 2021). Conversely, the company's income also reflects the company's ability to crowd source and innovate. Based on these considerations, this study takes the ratio of base city enterprise income to national enterprise income as an output variable (Y_1_) of the DEA model.

#### Urban GDP Ratio Y_2_

Since 1985, China has used GDP to calculate the national economy, and established the theoretical basis of the national economic accounting system. Urban GDP represents the level of economic development of a city and can measure the economic development status of a city, including the economic development status of enterprises. It is an important macroeconomic indicator. Based on these considerations, this study takes the ratio of base city GDP to national GDP as an output variable (Y_2_) of the DEA model.

## Data Analysis and Results

### Instrument

This research collects relevant data of 21 service outsourcing base cities across China from 2016 to 2019. Data sources: “Service Outsourcing Network,” “China Service Outsourcing Network,” “China City Statistical Yearbook,” “China City Competitiveness of each base city Report,” “China Service Outsourcing Development Report,” “China Statistical Yearbook,” as well as data from the statistical bureaus of base cities, data from government websites, enterprise data and related documents. These 21 cities include the capital Beijing and other major cities in China. These cities are very representative in Northeast China, North China, East China, South China and southwest China. Through the DEA software and model, 21 DMU data were input and calculated, and the results are shown in Table 1.

**Table 1:** Analysis results.

**DMU**	** ${\text{S}}_{1}^{-}$ **	** ${\text{S}}_{2}^{-}$ **	** ${\text{S}}_{3}^{-}$ **	** ${\text{S}}_{4}^{-}$ **	** ${\text{S}}_{5}^{-}$ **	** ${\text{S}}_{6}^{-}$ **	** ${\text{S}}_{1}^{+}$ **	** ${\text{S}}_{2}^{+}$ **	**vrstex**	**scale**	**θ**	**Rank**
Beijing	0.000	0.000	0.000	0.000	0.000	0.000	0.000	0.000	1.000	1.000	1.000	1
Tianjin	0.341	0.315	0.432	0.000	0.000	0.467	0.000	0.000	1.000	0.689	0.689	3
Shanghai	0.000	0.000	0.000	0.000	0.000	0.000	0.000	0.000	1.000	1.000	1.000	1
Chongqing	0.125	0.102	0.000	0.313	0.000	0.543	0.000	0.000	1.000	0.831	0.831	2
Dalian	0.000	0.000	0.226	0.234	0.068	0.152	0.000	0.000	0.437	1.000	0.437	10
Shenzhen	0.000	0.000	0.542	0.000	0.000	0.000	0.000	0.000	0.463	1.000	0.463	8
Guangzhou	0.000	0.156	0.337	0.331	0.105	0.000	0.000	0.000	0.510	1.000	0.510	7
Wuhan	0.000	0.167	0.121	0.116	0.000	0.000	0.000	0.000	0.385	1.000	0.385	11
Haerbin	0.000	0.000	0.452	0.000	0.000	0.223	0.000	0.000	1.000	0.546	0.546	5
Chengdu	0.000	0.000	0.235	0.000	0.096	0.150	0.000	0.000	1.000	0.580	0.580	4
Nanjing	0.000	0.000	0.000	0.000	0.000	0.000	0.000	0.000	1.000	1.000	1.000	1
Xi'an	0.000	0.152	0.313	0.246	0.000	0.221	0.000	0.000	0.366	0.435	0.310	14
Jinan	0.000	0.261	0.000	0.000	0.174	0.000	0.000	0.000	0.375	0.443	0.352	12
Hangzhou	0.000	0.000	0.000	0.000	0.000	0.000	0.000	0.000	1.000	1.000	1.000	1
Hefei	0.000	0.000	0.310	0.000	0.000	0.000	0.000	0.000	0.283	0.341	0.266	15
Nanchang	0.000	0.113	0.000	0.000	0.114	0.210	0.000	0.000	1.000	0.521	0.521	6
Changsha	0.000	0.054	0.000	0.000	0.000	0.112	0.000	0.000	0.383	0.421	0.340	13
Daqing	0.000	0.000	0.000	0.000	0.000	0.000	0.000	0.000	0.281	0.330	0.265	16
Suzhou	0.000	0.000	0.000	0.000	0.000	0.000	0.000	0.000	1.000	1.000	1.000	1
Wuxi	0.000	0.000	0.000	0.000	0.000	0.000	0.000	0.000	1.000	1.000	1.000	1
Xiamen	0.000	0.000	0.000	0.000	0.000	0.000	0.000	0.000	0.452	1.000	0.452	9

### Results Analysis

It can be seen from Table 1 that the values of θ, scale and vrste in the six base cities of Beijing, Shanghai, Nanjing, Hangzhou, Suzhou and Wuxi are all 1, indicating that crowd sourcing in these cities is effective for DEA, and their economic activities are both technological and The scale is effective, that is, the crowd sourcing performance of these 6 base cities is better, and the expected effect of input and output is achieved. The crowd sourcing competitiveness of these six cities in the 21 base cities is relatively strong, and crowd sourcing is basically known by enterprises and the public., with the help of crowd sourcing, it has brought expected innovation value to enterprises. Among these six cities, Beijing is the economic and political capital of China, and the remaining 5 cities are all located in the Yangtze River Delta economic belt. The degree of economic development is positively correlated. The five base cities of Xi'an, Jinan, Hefei, Changsha, and Daqing, the θ, scale, and vrste values are all not equal to 1, indicating that crowd sourcing in these cities is not effective in DEA, and the competitiveness of crowd sourcing is relatively weak, and their economic activities are also technical Compared with the scale, the efficiency is not high, and the expected effect of crowd sourcing of input and output has not yet been achieved. Enterprises, governments and other organizations can jointly improve the crowd sourcing competitiveness of these five base cities. First, enterprises should improve their own technical performance. Second, it is the government and other organizations that need to coordinate the layout and policy guidance and support. The third is to combine local advantages into the crowd sourcing model to try to develop with characteristics. The fourth is to improve the competitiveness of crowd sourcing by improving the input ratio or output ratio of resources.

The five cities of Tianjin, Chongqing, Harbin, Chengdu and Nanchang have a vrste value of 1, while the θ and scale values are not 1, indicating that the crowd sourcing input resources of these base cities have been fully utilized and have certain potential for crowd sourcing. competitiveness, but the overall technical performance has not yet reached the optimization of input and output. The methods for these five base cities to improve the competitiveness of crowd sourcing are as follows: first, to strengthen the demonstration effect of leading enterprises; second, to increase the effect of crowd sourcing industry clusters; third, to guide Enterprises achieve economies of scale, and then achieve optimal input and output. The five base cities of Dalian, Shenzhen, Guangzhou, Wuhan and Xiamen have a scale value of 1, while the θ and vrste values are not 1, indicating that the input and output of these cities are not optimal, which may be caused by pure technical performance. The competitiveness of crowd sourcing is weak, the government and industry may not pay enough attention to crowd sourcing, and the crowd sourcing atmosphere is not strong. The paths to improve the competitiveness of crowd sourcing are as follows: first, to increase the attention of the government and industry; second, to establish a gradual Perfect crowd sourcing incentive mechanism and evaluation preparation system; third, enterprises improve the overall technical efficiency of input resources.

Through the statistics and analysis of the data in Table 1, the DEA competitiveness statistics of each base city are obtained, as shown in Table 2.

**Table 2:** Results DEA competitiveness statistics.

**Type**	**Min**	**Max**	**Ave**	**Stdev**
θ	0.314	1.000	0.6313	0.2656
scale	0.383	1.000	0.7946	0.2383
vrste	0.314	1.000	0.7536	0.2789

It can be seen from Table 2 that the mean value of θ is 0.6313. It can be considered that nearly 37% of the resource input and output of each base city have not achieved the expected effect, and the overall crowd sourcing performance of each base city needs to be further improved. It is considered that without considering the ineffectiveness of pure technical efficiency, the ratio of the low crowd sourcing competitiveness of each base city due to factors such as its own scale is close to 21%; The ratio of cities with low crowd sourcing competitiveness due to inappropriate resource allocation is close to 25%. The mean values of Scale and vrste are not much different. It can be considered that the gap between the scale efficiency of crowd sourcing and the efficiency of resource allocation in 21 base cities in China is not very large. The Stdev values of θ, scale and vrste are not large, indicating that there is no significant difference in the crowd sourcing competitiveness among individuals in the 21 base cities.

## Discussions and Implications

### Research Discussions

The Internet 3.0 era is the era of service outsourcing, and more importantly, the era of crowd sourcing. Actively understanding and participating in crowd sourcing will enable enterprises to be at the forefront of innovation. With the help of data envelopment analysis method, this study organizes and analyzes the data of 21 service outsourcing base cities in China from 2016 to 2019, and uses input-output efficiency to explain the crowd sourcing competitiveness of service outsourcing base cities. Unfortunately, from the survey, we found that the overall trend of Chinese enterprises is that crowd sourcing is not very competitive, and the input and output have not been able to exert the maximum effect. Many enterprises have no strong awareness of participating in crowd sourcing. Local governments have not yet formed a crowd sourcing incentive mechanism.

### Implications

This study offers useful implications from three aspects. First, when adopting the crowd sourcing model, enterprises should consider the actual background of service outsourcing in their local area to avoid low-level investment of resources and vicious competition among enterprises.

Second, local governments should actively provide policy support and guidance, and rationally deploy long-term planning. In particular, the base cities where crowd sourcing is not effective for DEA should conduct in-depth analysis and broad thinking, and establish a positive crowd sourcing service evaluation system and incentive mechanism. It is also necessary to formulate a reasonable crowd sourcing system to standardize the order of crowd sourcing enterprises.

Third, give full play to the demonstration radiation effect of crowd sourcing enterprises in the Yangtze River Delta, the Pearl River Delta and the Bohai Sea Rim Economic Circle, form a crowd sourcing atmosphere and crowd sourcing industry clusters, learn from foreign advanced crowd sourcing experience, and combine Chinese economic development to explore suitable crowd sourcing in China. The “Chinese-style” crowd sourcing road for enterprise development.

### Research Limitations and Future Research

Finally, it should be pointed out that the literature review found that the research on crowd sourcing in academia is still in the exploratory stage, and there is a lack of quantitative analysis on the causes of crowd sourcing and related performance. Therefore, the research on crowd sourcing in this study is still very simple, the collected data still lacks a certain representativeness, and the selection of variables is still not comprehensive and convincing. There are still many studies in the field of crowd sourcing that have not been explored by scholars, such as the contractual relationship between crowd sourcing, crowd sourcing and human resource incentives, crowd sourcing intellectual property rights, crowd sourcing information security, and crowd sourcing model comprehensive management are still in a vacuum state. Scholars, including this research, should try from these aspects in future exploration.

## Data Availability Statement

The raw data supporting the conclusions of this article will be made available by the authors, without undue reservation.

## Author Contributions

XS designed the study and drafted the initial manuscript. YG, XZ, and JX collected the data, performed statistical analysis, and drafted the initial manuscript. YG contributed to the revised manuscript. All authors discussed the results and contributed to the final manuscript.

## Funding

This study was supported by the general project of the Jiangsu University Philosophy and Social Science Research Project (Project No. 2020SJA1408, leading researcher of the project group: XS).

## Conflict of Interest

The authors declare that the research was conducted in the absence of any commercial or financial relationships that could be construed as a potential conflict of interest.

## Publisher's Note

All claims expressed in this article are solely those of the authors and do not necessarily represent those of their affiliated organizations, or those of the publisher, the editors and the reviewers. Any product that may be evaluated in this article, or claim that may be made by its manufacturer, is not guaranteed or endorsed by the publisher.

## References

[B1] Brabham D. C. (2008). Crowdsourcing as a model for problem solving:an introduction and cases. Int. J. Res. New Media Technol. 14, 75–90. 10.1177/1354856507084420

[B2] Charnes A. Cooper W. W. Rhodes E. (1978). Measuring the efficiency of decision making units. Eur. J. Oper. Res. 6, 429-444. 10.1016/0377-2217(78)90138-8

[B3] Cuccolo K. Irgens M. S. Zlokovich M. S. Grahe J. Edlund J. E. (2020). What crowdsourcing can offer to cross-cultural psychological science. Cross Cult. Res. 55, 3–28, 10.1177/1069397120950628

[B4] Deichmann D. Gillier T. Tonellato M. (2021). Getting on board with new ideas: an analysis of idea commitments on a crowdsourcing platform. Res. Policy 50, 104320. 10.1016/j.respol.2021.104320

[B5] Dissanayake I. Nerur S. Wang J. Yasar M. (2021). The impact of helping others in coopetitive crowdsourcing communities. J. Assoc. Inf. Syst. 22, 67–101. 10.17705/1jais.00654

[B6] Donlon E. Costello E. Brown M. (2020). Collaboration, collation, and competition: Crowdsourcing a directory of educational technology tools for teaching and learning. Aust. J. Educ. Technol. 36, 41–55. 10.14742/ajet.5712

[B7] Dyckhoff H. Allen K. (2001). Measuring ecological efficiency with data envelopment analysis (DEA). Eur. J. Oper. Res. 132, 312–325. 10.1016/S0377-2217(00)00154-5

[B8] Eric V. H. (1988). The Source of Innovation. NewYork, NY: Oxford University Press.

[B9] Erica S. Tim W. Alderfer M. A. Karen A. R. N. Jennifer C. Allison K.. (2020). Topical review: crowdsourcing as a novel approach to qualitative research. J. Pediatr. Psychol. 46, 189–196, 10.1093/jpepsy/jsaa096 33236059 PMC7896271

[B10] Friedman T. (2006). The World is Flat. London: Penguin Books.

[B11] Füller J. Hutter K. Krger N. (2021). Crowdsourcing as a service – from pilot projects to sustainable innovation routines. Int. J. Project Manag. 39, 183–195. 10.1016/j.ijproman.2021.01.005

[B12] Gordon A. (2021). Crowdsourcing and its relationship to wisdom of the crowd and insight building: a bibliometric study. Scientometrics 126, 4373–4382. 10.1007/s11192-021-03932-z

[B13] Hou L. Hsueh S. C. Zhang S. (2020). Does formal financial development crowd in informal financing? evidence from chinese private enterprises. Econ. Model. 90, 288–301. 10.1016/j.econmod.2020.05.015

[B14] Howe J. (2006). Gannett to crowdsource news. Wired. 1–2.

[B15] Jaribion A. Khajavi S. H. Jarvihaavisto U. Nurmi I. Holmstrom J. (2021). ‘Crowdsourcing properties and mechanisms of mega hackathons: the case of junction,” in IEEE Transactions on Engineering Management, 1–15. 10.1109/TEM.2021.3079107

[B16] Li L. Y. Bensi M. Cui Q. B. Baecher G. B. Huang Y. (2021). Social media crowdsourcing for rapid damage assessment following a sudden-onset natural hazard event. Int. J. Inf. Manag. 60, 102378. 10.1016/j.ijinfomgt.2021.102378

[B17] Lim J. E. Lee J. Kim D. (2021). The effects of feedback and goal on the quality of crowdsourcing tasks. Int. J. Hum. Comput. Interact. 37, 1207–1219. 10.1080/10447318.2021.1876355

[B18] Mandera P. Keuleers E. Brysbaert M. (2020). Recognition times for 62 thousand English words: data from the English crowdsourcing project. Behav. Res. Methods. 52, 741–760. 10.3758/s13428-019-01272-8 31368025

[B19] Modaresnezhad M. Lakshmi lyer Palvia P. Taras V. (2020). Information Technology (IT) enabled crowdsourcing: A conceptual framework. Inf. Process. Manag. 57, 102135. 10.1016/j.ipm.2019.10213526425429

[B20] Nevo D. Kotlarsky J. (2020). Crowdsourcing as a strategic is sourcing phenomenon: critical review and insights for future research. J. Strat. Inf. Syst. 29, 101593. 10.1016/j.jsis.2020.101593

[B21] Ozcan S. Boye D. Arsenyan J. Trott P. (2020). A scientometric exploration of crowdsourcing: research clusters and applications. IEEE Trans. Eng. Manag. 99, 1–15. 10.1109/TEM.2020.3027973

[B22] Sawhney M. Prandell E. (2001). Communities of creation: management distributed innovation turbulent market. Calif. Manag. Rev. 42, 24–49. 10.2307/41166052

[B23] Shank D. B. (2016). Using crowdsourcing websites for sociological research: the case of amazon mechanical turk. Am. Sociol. 47, 47–55. 10.1007/s12108-015-9266-9

[B24] Shao B. J. Shi L. Xu B. Liu L. (2012). Factors affecting participation of solvers in crowdsourcing: an empirical study from China. Electron Markets 22, 73–82. 10.1007/s12525-012-0093-3

[B25] Shergadwala M. Forbes H. Schaefer D. Panchal J. H. (2020). Challenges and research directions in crowdsourcing for engineering design: an interview study with industry professionals. IEEE Trans. Eng. Manag. 99, 1–13. 10.1109/TEM.2020.2983551

[B26] Ullah A. Zhang Q. Ahmed M. (2021). The influence of intellectual property rights protection on contribution efforts of participants in online crowdsourcing contests. Comput. Hum. Behav. 123, 106869. 10.1016/j.chb.2021.106869

[B27] Welinder P. Perona P. (2010). Online crowdsourcing: Rating annotators and obtaining cost-effective labels, in 2010 IEEE Computer Society Conference on Computer Vision and Pattern Recognition – Workshops (San Francisco, CA). 10.1109/CVPRW.2010.5543189

[B28] Wu W. Gong X. (2021). Motivation and sustained participation in the online crowdsourcing community: the moderating role of community commitment. Internet Res. 31, 287–314. 10.1108/INTR-01-2020-0008

[B29] Xu H. Wu Y. Hamari J. (2022). What determines the successfulness of a crowdsourcing campaign: a study on the relationships between indicators of trustworthiness, popularity, and success. J. Bus. Res. 139, 484–495. 10.1016/j.jbusres.2021.09.032

[B30] Ziegler W. Lehner K. KommPas Study Group. (2021). Crowdsourcing as a tool in the clinical assessment of intelligibility in dysarthria: how to deal with excessive variation. J. Commun. Disord. 93, 106135. 10.1016/j.jcomdis.2021.106135 34214758

